# Rapamycin Treatment Improves Neuron Viability in an *In Vitro* Model of Stroke

**DOI:** 10.1371/journal.pone.0068281

**Published:** 2013-07-04

**Authors:** Lauren Fletcher, Teresa M. Evans, Lora Talley Watts, David F. Jimenez, Murat Digicaylioglu

**Affiliations:** 1 Department of Neurosurgery, University of Texas Health Science Center at San Antonio, San Antonio, Texas, United States of America; 2 Department of Cellular and Structural Biology, University of Texas Health Science Center at San Antonio, San Antonio, Texas, United States of America; Sanford-Burnham Medical Research Institute, United States of America

## Abstract

Ischemic stroke is the leading cause of serious, long-term adult disability and is associated with sensorimotor and cognitive impairments due to neuronal degeneration. Currently, recombinant tissue plasminogen activator (rTPA) is the only FDA-approved medical therapy for treatment of patients with acute ischemic stroke. However, rTPA can only be given within 3 hours of symptom onset, and only 2% of patients are eligible. Therefore, there is an urgent need for novel neuroprotective treatment options for ischemic stroke. An emerging treatment for a diverse range of neurological disorders associated with neurodegeneration is rapamycin, a key modulator of the mammalian target of rapamycin (mTOR) pathway. The mTOR pathway is the primary regulator of the cellular response to nutrient availability, changes in energy status and stress as seen following ischemia and reperfusion. However, rapamycin’s effects on mTORC1 and mTORC2 are poorly understood in neurons. In the current study we show that rapamycin can prevent the activation of both mTORC1 and mTORC2 in cortical neurons and improve cell survival following oxygen glucose deprivation (OGD), an in vitro model of ischemic stroke. This work further supports the investigation of rapamycin as a novel neuroprotectant for ischemic stroke.

## Introduction

Stroke is the fourth leading cause of death in the United States resulting in dramatic neurological impairments and decreased quality of life [1]. There is an urgent need for novel neuroprotective treatment options for ischemic stroke, which affects 795,000 people and results in an estimated yearly cost of over $73.7 billion (2010) [2]. Currently, thrombolysis is the only FDA approved treatment. However, treatment delays, a narrow therapeutic window (3 to 4.5 hours after the onset of symptoms) and pre-existing co-morbidities disqualify 98% of patients from thrombolysis [3]. The ultimate goal of a neuroprotective strategy for stroke is to maintain adequate brain function and neurological ability following injury associated with ischemia and reperfusion. Currently, treatments aiming to achieve ischemic neuroprotection use multiple treatment modalities such as N-methyl-D-aspartate (NMDA) receptor antagonists, calcium channel blockers and antioxidants for management of stroke but none have been able to significantly reverse neuronal damage following both ischemia and reperfusion injury [4].

An emerging treatment for a diverse range of neurological disorders associated with neurodegeneration is rapamycin, a key modulator of the mammalian Target of Rapamycin (mTOR) pathway. The mTOR pathway is the primary regulator of the cellular response to nutrient availability, changes in energy status and stress as seen following ischemia and reperfusion [5]. Treatment with rapamycin promotes neuronal viability and reduces neurological damage in multiple animal CNS injury models[6]–[11].

The current study investigates the effects of rapamycin on mTOR signaling and neuron survival in an *in vitro* model of ischemic stroke using oxygen glucose deprivation (OGD). OGD induces metabolic and oxidative stress, excitoxicity, apoptosis, and inflammatory processes comparable to that associated with ischemic stroke [12]. Conversely, this model also mimics the changes in the cellular environment following reperfusion (reoxygenation), the primary result of reperfusion after transient occlusions in animal models and rTPA mediated thrombolysis, the most widely used treatment for stroke patients [13]. Reperfusion returns the affected neuronal region to normal energy and normoxic conditions by restoring blood flow to the infarcted area which is sufficient to activate the mTOR pathway [12] ^,^ [14].

mTOR is activated by phosphorylation at multiple sites (Ser-2448, Ser-2481, Thr-2446, and Ser-1261), with Ser-2448 and Ser-2481 being most critical for kinase activity [5], [15], [16]. Additionally, phosphorylation of mTOR regulates the formation of two major heteromeric and functionally distinct complexes: mTOR Complex 1 (mTORC1) and mTOR Complex 2 (mTORC2), with mTORC1 predominantly containing mTOR phosphorylated on Ser-2448 and mTORC2 predominantly containing mTOR phosphorylated on Ser-2481 [17]. These two complexes are characterized by their specific binding proteins raptor and rictor. Raptor is an essential scaffolding protein for the formation of mTORC1. In a similar fashion mTORC2 is bound by rictor [15], [18], [19]. Functionally, raptor and rictor serve to enhance substrate specificity of mTOR towards its downstream targets, p70 ribosomal S6 Kinase (p70S6K) and Akt respectively [5].

The primary function of mTORC1 is to directly regulate protein synthesis in response to intracellular and extracellular stress and changes in nutrient availability, as in ischemia and reperfusion [16]. Under conditions of low nutrient and oxygen availability mTORC1 decreases protein synthesis, neuron growth and proliferation, and promotes autophagy, a physiological process whereby a neuron selectively destroys intracellular waste products [15], [18], [19]. mTORC1 is reciprocally phosphorylated at Ser-2448 by its down stream target, p70S6K [20]. Phosphorylation of p70S6K by mTOR is down-regulated in response to decreased amino acid availability and rapamycin treatment. Through the subsequent inhibition of its downstream target p70S6K, mTORC1 decreases protein synthesis, cellular growth and autophagy [20], [21].

Two main functions of mTORC2 have been characterized. The first is its role in maintaining cytoskeleton integrity. Second, in conjunction with PDK1 phosphorylation of Akt at Threonine 308, mTORC2 initiates the phosphorylation and activation of Akt at Ser-473. Subsequently, Akt promotes neuron proliferation, survival, and migration, partly through promoting mTORC1 activity. Thus, Akt connects mTORC1 to mTORC2 signaling [15], [18], [19]. The activation of Akt has been reported to be associated with improved neuronal outcome in multiple models of stroke[22]–[25].

Rapamycin binds to its intracellular receptor FK-binding protein 12 (FKBP12) and the resulting complex interacts with the FKBP12-rapamycin binding (FRB) domain located in the C-terminus of mTOR [26]. Binding of the rapamycin/FKBP12 complex to the FRB domain of mTOR inhibits the interaction of raptor with mTOR, thereby reducing raptor-dependent mTOR substrate phosphorylation of p70S6K [27], [28]. Since p70S6K is believed to be the main 2448 kinase for mTOR, rapamycin inhibition of p70S6K leads to a decrease in the phosphorylation of mTOR at Ser-2448 [17]. In contrast to mTORC1, mTORC2 does not bind rapamycin/FKBP12 and this is thought to confer mTORC2 its resistance to acute rapamycin treatment [29]. However, it has been shown that mTORC2 is sensitive to prolonged rapamycin treatment in certain cell types in which rapamycin prevents the formation of the mTORC2 complex [17], [30], and it has been suggested that rapamycin is actually a cell-type-dependent inhibitor of mTORC2, whereas rapamycin is a universal inhibitor of mTORC1 [30].

Rapamycin’s effects on mTORC1 and mTORC2 are poorly understood in neurons. Therefore, this study assesses the effects of rapamycin on mTOR signaling and neuronal survival in an *in vitro* model of ischemic stroke, OGD. Following both OGD and rapamycin treatment we have shown changes in the localization of mTOR and in its activation state. We have also shown congruent changes in the activation of the down stream targets Akt and p70S6K. Furthermore, we demonstrate beneficial effects of rapamycin treatment on neuronal viability following OGD.

## Results

### Inhibition of mTOR Phosphorylation by Rapamycin

**Figure 1:**
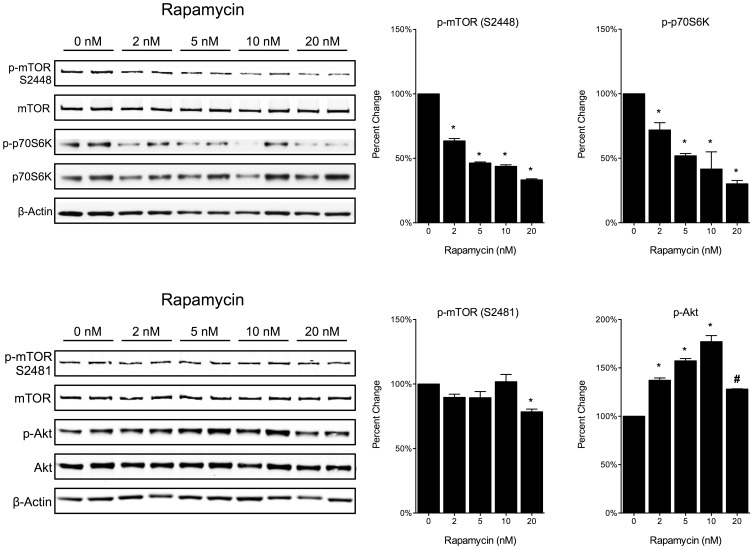
Rapamycin suppresses mTOR phosphorylation at Ser-2448 in a dose dependent manner. Primary cortical neurons were treated under normoxic conditions with various concentrations of rapamycin (0 nM – 20 nM). (A,B) Rapamycin caused a dose-dependent decrease in phosphorylated mTOR at Ser-2448, and subsequently caused a dose-dependent decrease in phosphorylated p70S6K. (C,D) Rapamycin at 20 nM caused a small decrease in phosphorylated mTOR at Ser-2481. The lower concentrations of rapamycin (2 nM – 10 nM) increased the phosphorylation of Akt, whereas 20 nM of rapamycin caused a decrease in phosphorylated Akt. (A,B) Representative immunoblots. (B,D) Quantitative analysis is shown as percent control for all phospho-proteins normalized to total protein. Data is represented as the mean ± s.e.m. (n = 4). *p<0.05 vs. 0 nM, #p<0.05 10 nM vs. 20 nM.

In order to better understand rapamycin’s effects on mTORC1 and mTORC2 in primary cortical neurons, cell cultures were treated with multiple doses of rapamycin (0 nM, 2 nM, 5 nM, 10 nM, and 20 nM) for 1.5 h. Western blot analysis shows that rapamycin is able to decrease phosphorylation of mTOR at Ser-2448 and subsequently, phosphorylation of p70S6K, in a dose dependent manner (Fig 1A,B). We also found that 20 nM of rapamycin was able to cause a small but significant reduction (21.6% ± 2.1%) in phosphorylated mTOR at Ser-2481 (Fig 1C,D). Interestingly, rapamycin at the lower doses (2, 5 and 10 nM) increased phosphorylation of Akt (Fig 1C,D). At 20 nM, however, phosphorylated Akt drops significantly, which may be due to the decrease in phosphorylated mTOR at Ser-2481.

### Rapamycin Improved Neuronal Viability Following OGD

**Figure 2:**
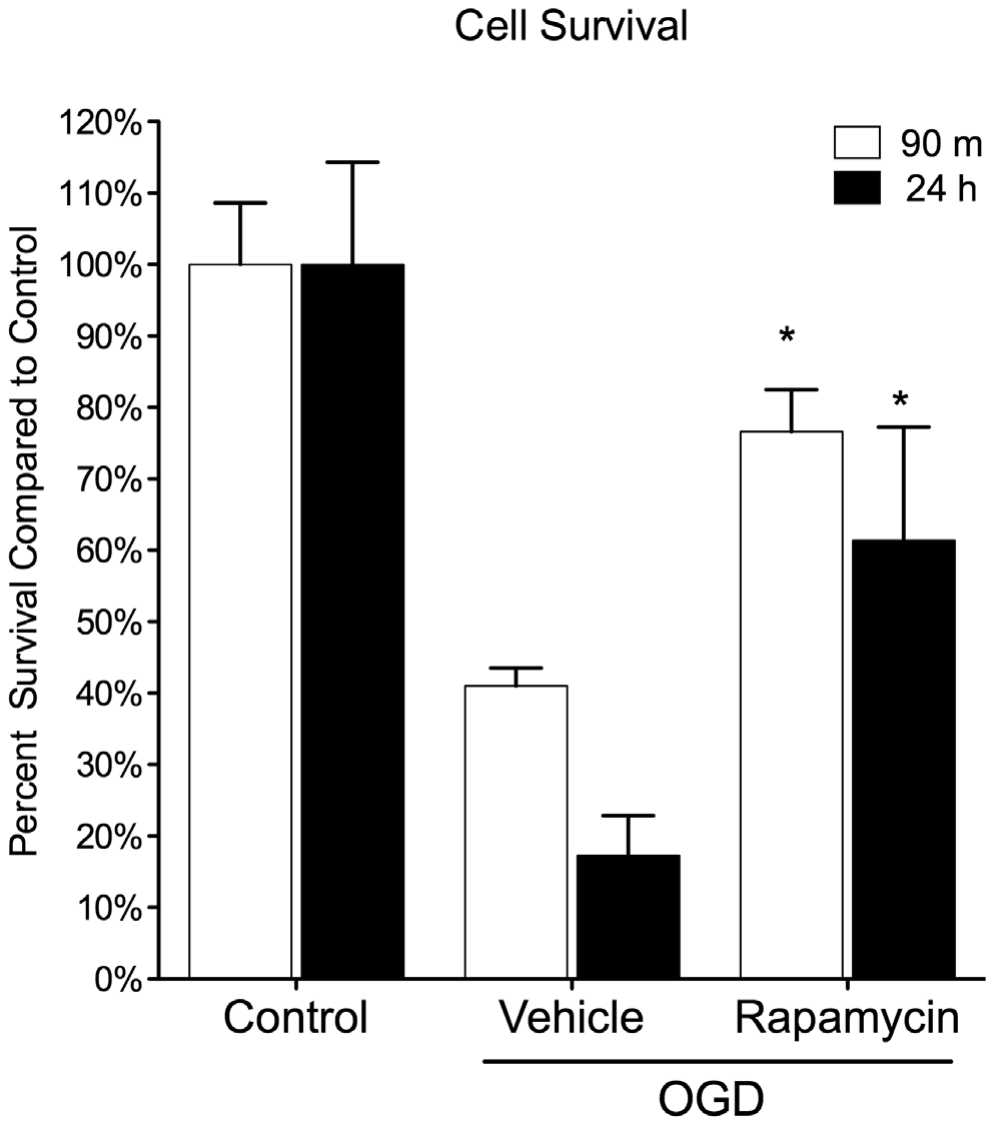
Rapamycin treatment increases cell survival following OGD. Primary cortical neurons were subjected to 1 hour OGD and then treated with rapamycin (20 nM) or vehicle (0.01% EtOH) for 90 m and 24 h. Cultures were immunostained with Hoechst (blue, nucleus stain) and MAP-2 (red, neuronal marker). MAP-2-positive cells were counted in 5 random fields on each of three cover slips per condition. Rapamycin significantly improved cell survival at both 90 m and 24 h following OGD when compared to vehicle. Data is represented as the mean ± s.e.m. (n = 3). *p<0.05 rapamycin vs. vehicle.

Immunofluorescence was used to determine the beneficial effects of rapamycin on neuronal viability and morphology following OGD (Fig 2). Neurons subjected to 1 h of OGD were treated with rapamycin (20 nM) or vehicle (0.01% EtOH), and then fixed after 90 m or 24 h. The number of viable neurons in the rapamycin-treated groups was significantly higher at 90 m (76.6% ± 5.9%) and 24 h (61.4% ± 14.2%) than in the vehicle-treated groups (41% ± 2.5% and 17% ± 5.6% respectively). Therefore, rapamycin seems to have a neuroprotective effect that is fast acting (prevents cell death after only 90 m) and long-lasting (protects up to 24 h).

**Figure 3:**
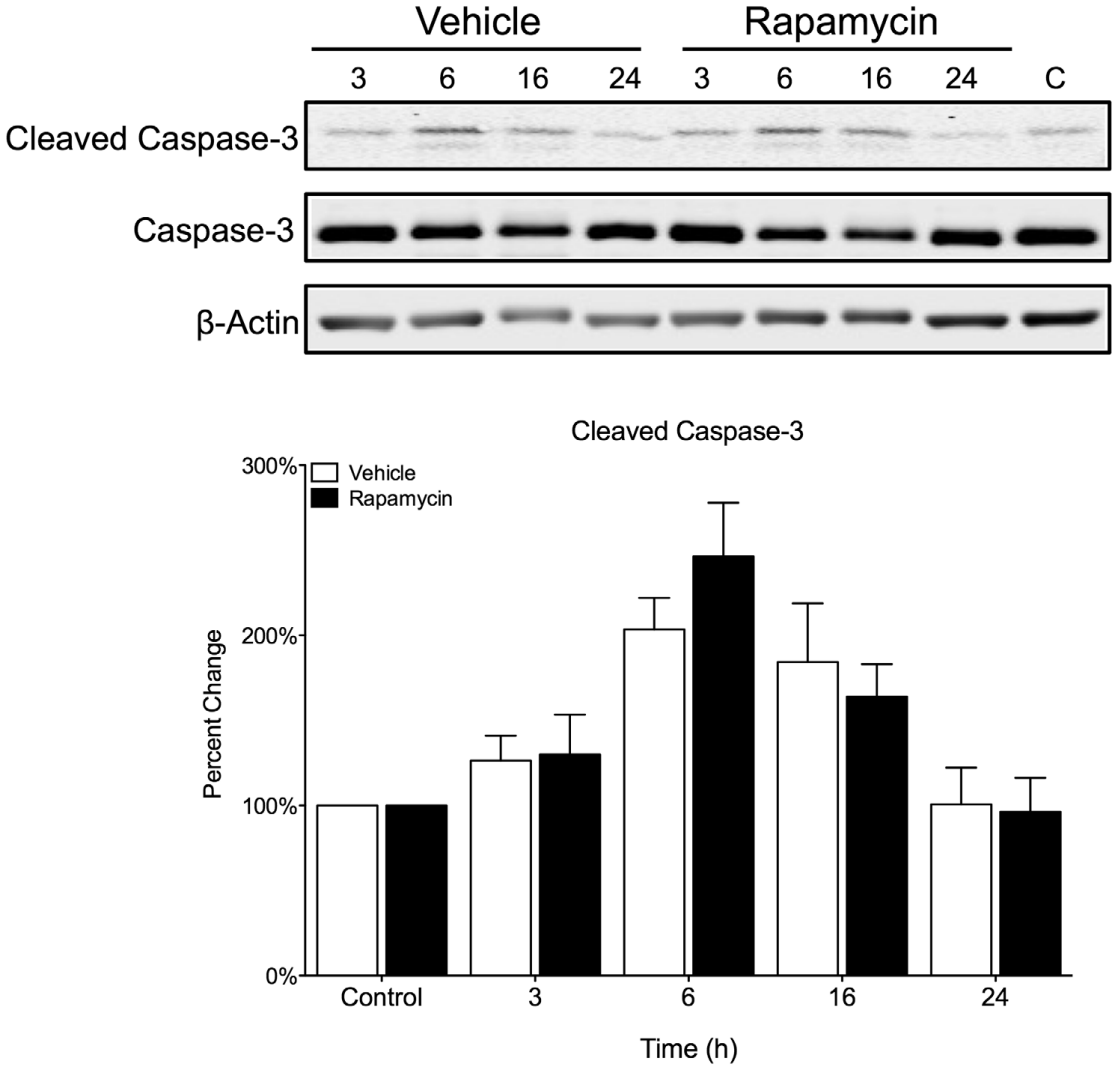
Rapamycin has no effect on caspase-3 in ischemic neurons. Primary cortical neurons were subjected to 1 h OGD and then treated with rapamycin (20 nM) or vehicle (0.01% EtOH) for multiple time points (3 h – 24 h). OGD for 1 h caused an increase in caspase-3 cleavage that peaked after 6 h. However, rapamycin had no effect on caspase-3 cleavage when compared to vehicle. (A) Representative immunoblots. (B) Quantitative analysis is shown as percent control for cleaved-caspase-3 normalized to caspase-3. Data is represented as the mean ± s.e.m. (n = 3).

Caspase-3 has been identified as a key mediator of apoptosis in animal models of ischemic stroke [31]. Caspase-3 cleaves many substrate proteins, including poly (ADP-ribose) polymerase (PARP), which leads to DNA injury and subsequently to apoptotic cell death. Due to the large increase in surviving cells, we checked to see if there was any effect on caspase-3 activation by rapamycin. We found that 1 h of OGD caused an increase in caspase-3 cleavage that peaked after 6 h (Fig 3). However, rapamycin had no effect on caspase-3 cleavage when compared to vehicle.

### Rapamycin Decreases Phosphorylated mTOR Following OGD

**Figure 4:**
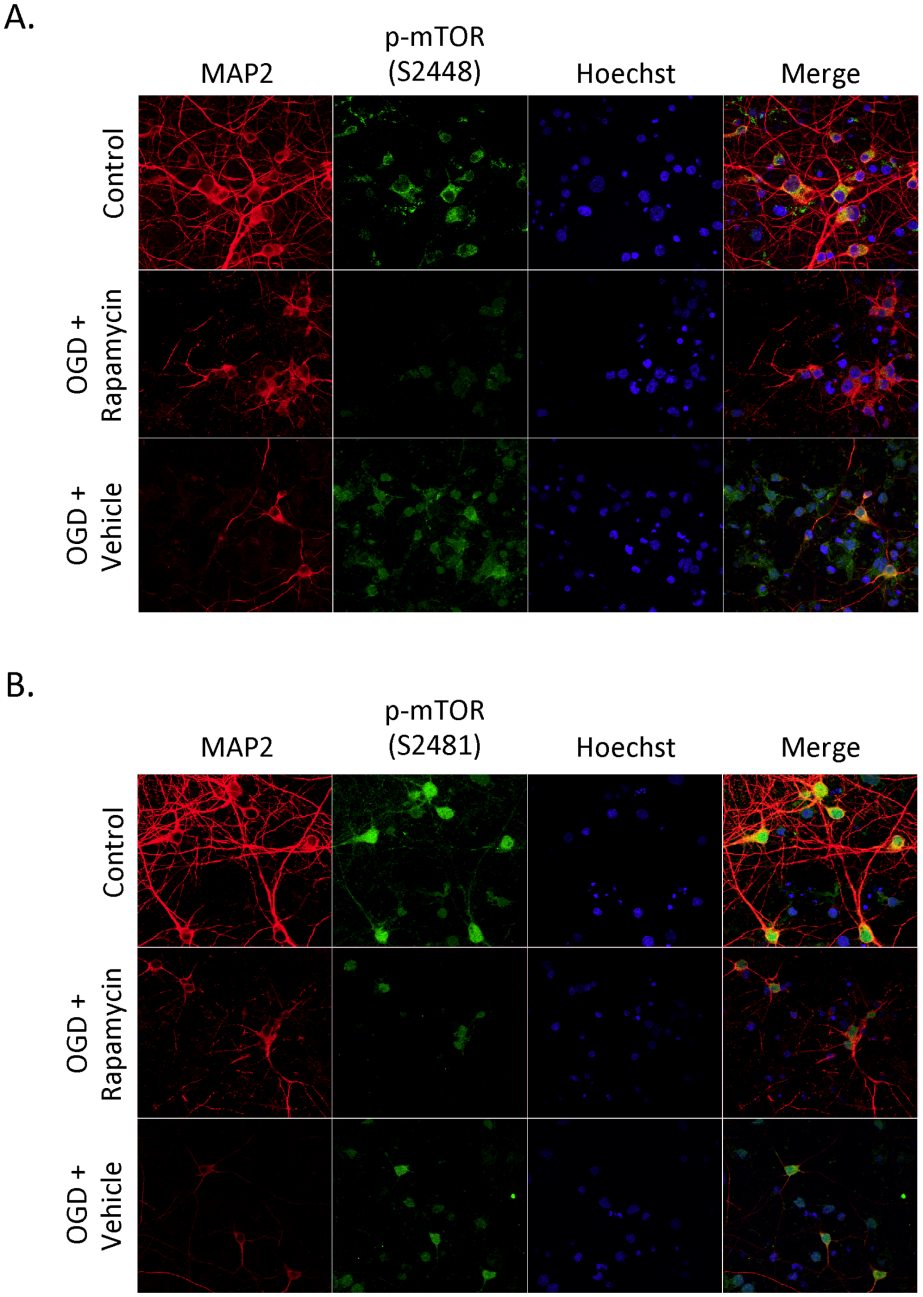
Rapamycin decreases phosphorylated mTOR following OGD. Primary cortical neurons were subjected to 1 hour OGD and then treated with rapamycin (20 nM) or vehicle (0.01% EtOH) for 24 h. (A) Cells were immunostained with phospho-mTOR Ser-2448 (green), Hoechst (blue) and MAP-2 (red). Under control conditions, Ser-2448 is mainly localized in the cytoplasmic domain. Under OGD conditions, rapamycin decreased the amount of Ser-2448 immunoreactivity. (B) Cells were immunostained with phospho-mTOR Ser-2481 (green), Hoechst (blue) and MAP-2 (red). Under control conditions, Ser-2481 is seen throughout the cell body, including the cytoplasm, nucleus and cell extensions. Under OGD conditions, rapamycin decreased the amount of Ser-2481 immunoreactivity. Images are representative figures (n = 3).

In order to further determine the effects of rapamycin treatment on phosphorylated mTOR at Ser-2448 and Ser-2481 after an ischemic event, we performed immunofluorescence on primary neuron cultures that had been subjected to OGD for 1 h and rapamycin treatment for 24 h. Under control conditions, phosphorylated mTOR at Ser-2448 is predominately expressed in the cytoplasmic region of the cell (Fig 4A). Phosphorylated mTOR at Ser-2481 is expressed throughout the cytoplasm, the nucleus and the cell extensions (Fig 4B). Twenty-four hours after OGD, rapamycin causes a significant decrease in both phosphorylated mTOR at Ser-2448 and Ser-2481 compared to vehicle (Fig 4A, B).

### Following OGD Rapamycin Reduces Phosphorylation of mTOR at Ser-2448 and Ser-2481

**Figure 5:**
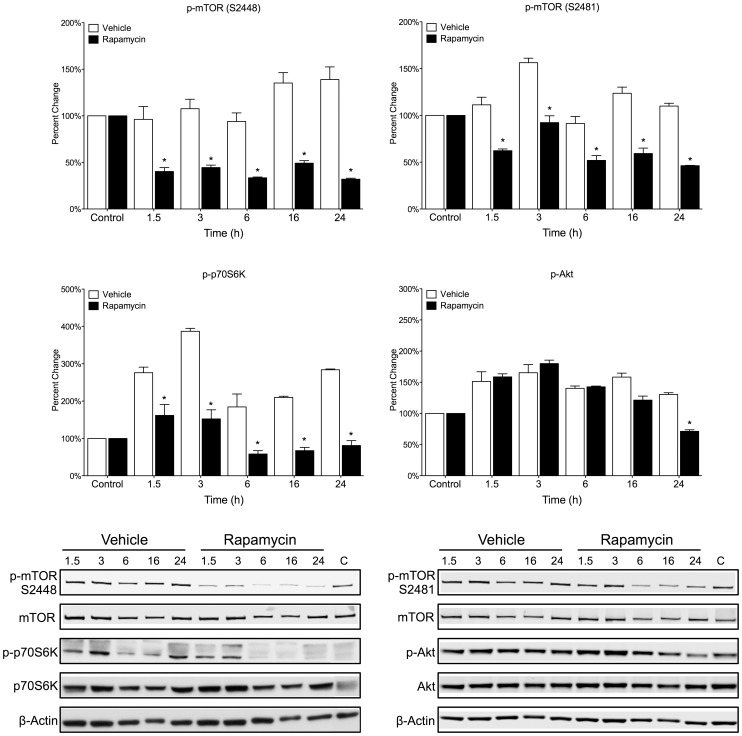
Following OGD mTOR phosphorylation is reversed by rapamycin treatment. Primary cortical neurons were subjected to 1 h OGD and then treated with rapamycin (20 nM) or vehicle (0.01% EtOH) for multiple time points (1.5 h–24 h). (A,B) Following OGD, rapamycin significantly decreased the phosphorylation of mTOR at both Ser-2448 and Ser-2481 compared to vehicle for all time points. (C,D) Following OGD, rapamycin significantly decreased the phosphorylation of p70S6K, the downstream target of mTORC1. However, rapamycin only caused a small decrease in phorphorylated AKT after 24 h. Rapamycin had no effect at any other time point. (A-D) Quantitative analysis is shown as percent control for all phospho-proteins normalized to total protein. Data is represented as the mean ± s.e.m. (n = 3). *p<0.05 vs. vehicle. (E,F) Representative immunoblots.

Using western blot analysis, we determined the phosphorylation state of mTOR at both Ser-2448 and Ser-2481 in primary neuron cultures following 1 h of OGD and in the presence of rapamycin (20 nM) or vehicle (0.01% EtOH) for various time points (1.5 – 24 h). Following OGD, rapamycin treatment for 1.5 h significantly decreases phosphorylated mTOR at Ser-2448 by 59.7% ± 4.2% (Fig 5A, E) and Ser-2481 by 47.7% ± 2.0% (Fig 5B, F) compared to the control. This decrease in phosphorylation is still significant after 24 h, where Ser-2448 has decreased by 78.0% ± 1.1% and Ser-2481 has decreased by 63.7% ± 0.3%.

mTORC1 activity can be monitored by p70S6K phosphorylation at Ser-371 [20]. Phosphorylation of p70S6K following OGD and vehicle treatment was significantly increased at 1.5 h and 3 h compared to control (Fig 5C, E). Rapamycin treatment decreased p70S6K phosphorylation significantly at all time points compared to vehicle. There was no significant change in total p70S6K levels. The decrease in phosphorylation of p70S6K following rapamycin treatment correlates with the decrease in phosphorylated mTOR at Ser-2448.

mTORC2 is responsible for the phosphorylation of Akt at Ser-473 [32]. We found that phosphorylation at this site is moderately induced in response to OGD in both vehicle and rapamycin treated groups and that rapamycin and vehicle treated groups are not significantly different at any of the time points tested between 1.5 and 16 h (Fig 5D, F). However, rapamycin does cause a small decrease in pAKT (28.7% ± 2.4%) after 24 h when compared to vehicle. Total Akt levels remained unchanged as compared to control.

## Discussion

The current study provides support for further investigation of rapamycin as a neuroprotective treatment modality for ischemia/reperfusion injury, such as stroke. We have characterized the effects of rapamycin treatment on mTOR signaling following 1 h of OGD and demonstrate that rapamycin treatment leads to a significant increase in neuronal viability *in vitro*.

The activation of mTORC1 and mTORC2 can be determined by measuring the phosphorylation of mTOR at Ser-2448 and Ser-2481 respectively [17]. This study establishes a dose dependent decrease in mTOR phosphorylation at Ser-2448 by rapamycin in primary cortical neurons *in vitro*. Our results indicate that in primary cortical neurons the concentration of rapamycin necessary to elicit a 50% reduction in Ser-2448 phosphorylation is 5 nM (Fig 1). The concentration of rapamycin used in the current study (20 nM) was sufficient for an approximate 70% reduction in Ser-2448 phosphorylation. Other studies, in multiple cell types, have used higher doses (up to 100 nM) of rapamycin to achieve similar reductions in mTOR phosphorylation. The current results indicate that in primary neurons the use of higher concentrations is not necessary and that the efficacy of rapamycin to inhibit mTOR phosphorylation at Ser-2448 is high at low nanomolar concentrations. Further studies to assess the effects of higher concentrations on cell viability in multiple cell types are necessary in order to confirm that the concentration of rapamycin being used is appropriate and not detrimental.

In contrast to mTORC1, mTORC2 has been shown to be resistant to acute rapamycin treatment [29]. However, previous studies have shown that rapamycin can inhibit the formation of mTORC2 in certain cell types by binding to free mTOR and preventing the further binding of Rictor [17], [30]. In this study, we found that 20 nM of Rapamycin for 1.5 h can cause a small but significant decrease (21.6% ± 2.1%) in phosphorylation of mTOR at Ser-2481 in primary cortical neurons. Additionally, we found that prolonged rapamycin treatment (up to 24 h) following OGD can cause a significant drop in Ser-2481 phosphorylation (63.7% ± 0.3%). Our data further supports the theory that rapamycin is a cell-type-dependent inhibitor of mTORC2 [30].

Under normoxic conditions immunofluorescent staining revealed that mTORC1 and mTORC2 are differentially expressed in neurons. Phosphorylated mTOR at Ser-2448 (mTORC1) is predominately expressed in the cytoplasm, whereas phosphorylated mTOR at Ser-2481 (mTORC2) is expressed throughout the cell body, including the cytoplasm, nucleus and cell extensions (Fig 4). A previous study in human cell lines has also demonstrated a similar expression pattern, in which mTORC1 was found in the cytoplasm and mTORC2 in the cytoplasm and nucleus [33].

In multiple models of neurodegenerative disease such as Alzheimer’s and Parkinson’s Diseases, inhibition of mTOR phosphorylation by rapamycin is correlated with improved neuronal viability [10], [34], [35]. In this study, we show following 1 h of OGD, that there is a significant increase in the number of neurons in the rapamycin-treated cultures compared to the vehicle-treated cultures (Fig 2), which further supports the use of rapamycin as a neuroprotectant following ischemia/reperfusion injury. Caspase-3 has been identified as a key mediator of apoptosis in animal models of ischemic stroke in vivo [31]. However, we found that rapamycin had no effect on caspase-3 cleavage when compared to vehicle in our OGD model *in vitro* (Fig 3). Therefore, rapamycin’s neuroprotective effects do not seem to involve the inhibition of caspase-3.

Following OGD, phosphorylated mTOR Ser-2448 is moderately increased in vehicle treated primary neuron cultures, which is significantly reversed with rapamycin treatment. Furthermore, p70S6K phosphorylation at Ser-371 is decreased with rapamycin treatment in correlation with the decrease in mTOR Ser-2448 phosphorylation (Fig 5). As a downstream target of mTORC1, the resulting decrease in p70S6K phosphorylation following rapamycin treatment indicates decreased mTORC1 signaling activity, which could potentiate cell viability through the reduction in protein synthesis, preservation of cellular nutrient stores and the release of autophagy inhibition. These results are congruent with previous work that indicates the phosphorylation of p70S6K is moderately increased in response to oxidative stress but can be decreased following a reduction in mTORC1 activity by rapamycin treatment [16], [36]. Decreases in the phosphorylation of mTOR at Ser-2448 and p70S6K at Ser-371 after treatment with rapamycin supports rapamycin’s ability to inhibit mTORC1 formation. The current and previous data indicate that a reduction in mTORC1 formation following injury results in improved neuronal viability.

Rapamycin treatment decreases Ser-2481 phosphorylation as compared to the vehicle treated group (Fig 5). This decrease does not clearly correlate with Akt phosphorylation at Ser-473, a key indicator of mTORC2 activity. Akt phosphorylation is moderately increased in both vehicle and rapamycin treated groups following OGD, which could play a role in the innate defense mechanism of the cell following OGD, possibly through the reduction of pro-apoptotic signaling. It is thought that mTORC2-dependent phosphorylation of Akt can be affected in one of three neuron type dependent ways following treatment with rapamycin: (1) Akt phosphorylation can be strongly inhibited (2) partially inhibited or (3) increased [30]. In our primary cortical neurons, rapamycin treatment (20 nM) had very little effect on Akt phosphorylation. Previous work indicates that following central nervous system injury such as ischemia, Akt activity is increased and becomes deregulated due to increased stimulation as a result of increased extracellular ATP. The current results indicate a moderate increase in Akt further supporting these findings [37], [38] [8], [39]. The increase in Akt activity could improve neuronal viability through increased inhibition of pro-apoptotic mediators and increased mitochondrial mediated glycolysis [40]–[42]. Further studies are needed to accurately elucidate the role of mTORC2/Akt signaling following OGD and reoxygenation such as the direct modulation of mTORC2 formation by knockdown of its associated protein Rictor.

The results of the current study suggest that rapamycin is an inhibitor of mTORC1 and mTORC2 in primary cortical neurons following OGD. We hypothesize that through the modulation of down-stream targets of mTOR, rapamycin promotes neuronal viability by decreasing protein synthesis, inhibiting pro-apoptotic factors and increasing autophagy. Determining the direct effects of rapamycin on mTORC1 and mTORC2 signaling will provide further understanding to the link between mTOR inhibition and improved neuronal viability. This study supports the continued research of mTOR modulation as a potential target for the development of novel treatments for ischemia/reperfusion injury in both *in vitro* and *in vivo* models of stroke.

## Materials and Methods

### Ethics Statement

All the animal work was carried out in the animal facility at the University of Texas Health Science Center in San Antonio (UTHSCSA) after being approved by the Animal Care and Use Committee at the UTHSCSA (Protocol Number 07071) and adhered to the National Institute of Health principles of laboratory animal care (NIH publication No. 80–23).

### Neuron Culture and Treatments

Neuronal cultures were harvested from embryonic day 17 Sprague-Dawley rats. Neurons were plated onto poly-L-lysine coated cover slips or 35 mm dishes and maintained in serum-free Neurobasal medium supplemented with B27 at 37°C, and 5% CO_2_ in a humidified environment. Neurons were incubated for a minimum of 14 days before experimentation.

Neurons were treated with oxygen glucose deprivation (OGD), 1 h in an environment containing 1% oxygen and glucose free media. Glucose free media was replaced with preconditioned media containing 20 nM rapamycin in 0.01% EtOH immediately following OGD treatment. Neurons were then harvested for use in western immunoblotting or immunofluorescence analysis.

### Western Immunoblotting Analysis

Western Blot Analysis was performed on neuron lysates at multiple time intervals following 1 h of oxygen glucose deprivation (OGD). Neurons were lysed in ice-cold lysis buffer with appropriate protease inhibitors (Neuron Signaling, Massachusetts, USA). Samples were centrifuged (14,000 rpm, 10 min, 4°C) to obtain supernatant and their protein concentration determined using bichoninic acid protein assay (Pierce, Illinois, USA). Equivalent amounts of protein (10 µg) from each sample were subjected to sodium dodecyl sulfate-polyacrylamide electrophoresis using 4–12% Bis–Tris gels (Invitrogen, California, USA) under reducing conditions and electro-blotted onto a nitrocellulose membrane. Following a blocking step (0.1% Tween-20/5% nonfat milk in PBS, 1 h, RT) membranes were incubated with primary antibodies overnight at 4°C with gentle agitation. The following primary antibodies were used (1∶1000, Neuron Signaling, Danvers, Mass., USA): phosphorylated AKT (Ser-473), phosphorylated mTOR (Ser-2448), phosphorylated mTOR (Ser-2481), mTOR (7C10), phosphorylated p70S6K (Ser-371), p70S6K, cleaved-caspase-3, caspase-3, β-Actin. Membranes were washed, and incubated with IRDye800 conjugated anti-rabbit IgG or IRDye680 conjugated anti-mouse IgG (LI-Cor Biosciences, Lincoln, NE). All antibody dilutions were made in non-mammalian Odyssey blocker (LI-Cor). Signal intensities were analyzed using the Odyssey infrared imaging system (LiCor).

### Immunofluorescence and Neuron Counting

Neurons were fixed in 4% paraformaldehyde for 15 min at RT, permeabilized (0.1% Tween-20 in PBS, 5 min), and unspecific binding of antibodies blocked with PBS/5.0% BSA for 1 h. Neurons were probed with primary antibodies and incubated overnight at 4°C. The following antibodies were used: (1∶500, Neuron Signaling, Danvers, Mass., USA): Anti-phosphorylated mTOR (Ser-2448), Anti-phosphorylated mTOR (Ser-2481), anti-MAP2. After a washing step (PBS, 5 min), neurons were incubated with AlexaFluor-conjugated secondary antibodies (1∶1000, 1 h, RT, PBS/5% BSA; Molecular Probes, Eugene, Oregon, USA). The nucleus was counterstained with Hoechst 33342 (Molecular Probes). Coverslips were then mounted onto slides with Prolong Gold Antifade reagent (Molecular Probes). Stained neurons were visualized with an Olympus FV-1000 confocal microscope (Olympus America Inc., Center Valley, Pennsylvania, USA) and images captured with FluoView v. 5.0 software (Olympus America Inc.).

For cell survival experiments, neurons were counted for each treatment condition on three separate cover slips from the same culture. Five fields were chosen randomly on each cover slip and neurons that were positively stained for MAP-2 and Hoechst were counted within each field. Values were then averaged and normalized to control.

### Statistical Analysis

Results are expressed as mean ± s.e.m. Statistical analysis was performed using one-way analysis of variance followed by a Tukey’s multiple comparison test. *P* < 0.05 was considered to be statistically significant.

## References

[1] Towfighi A, Saver JL (2011) Stroke declines from third to fourth leading cause of death in the United States: historical perspective and challenges ahead. Stroke 42: 2351–2355. 21778445 10.1161/STROKEAHA.111.621904

[2] Lloyd-Jones D, Adams RJ, Brown TM, Carnethon M, Dai S, et al (2010) Executive summary: heart disease and stroke statistics–2010 update: a report from the American Heart Association. Circulation 121: 948–954. 20177011 10.1161/CIRCULATIONAHA.109.192666

[3] Adams H, Adams R, Del Zoppo G, Goldstein LB (2005) Guidelines for the early management of patients with ischemic stroke: 2005 guidelines update a scientific statement from the Stroke Council of the American Heart Association/American Stroke Association. Stroke 36: 916–923. 15800252 10.1161/01.STR.0000163257.66207.2d

[4] Adams HP Jr (2001) Treatment of acute ischemic stroke: selecting the right treatment for the right patient. Eur Neurol 45: 61–66. 11244267 10.1159/000052097

[5] Foster KG, Fingar DC (2010) Mammalian target of rapamycin (mTOR): conducting the cellular signaling symphony. J Biol Chem 285: 14071–14077. 20231296 10.1074/jbc.R109.094003 PMC2863215

[6] Hailer NP (2008) Immunosuppression after traumatic or ischemic CNS damage: it is neuroprotective and illuminates the role of microglial cells. Prog Neurobiol 84: 211–233. 18262323 10.1016/j.pneurobio.2007.12.001

[7] Menzies FM, Rubinsztein DC (2010) Broadening the therapeutic scope for rapamycin treatment. Autophagy 6: 286–287. 20081360 10.4161/auto.6.2.11078

[8] Carloni S, Girelli S, Scopa C, Buonocore G, Longini M, et al (2010) Activation of autophagy and Akt/CREB signaling play an equivalent role in the neuroprotective effect of rapamycin in neonatal hypoxia-ischemia. Autophagy 6: 366–377. 20168088 10.4161/auto.6.3.11261

[9] Wouters BG, Koritzinsky M (2008) Hypoxia signalling through mTOR and the unfolded protein response in cancer. Nat Rev Cancer 8: 851–864. 18846101 10.1038/nrc2501

[10] Caccamo A, Majumder S, Richardson A, Strong R, Oddo S (2010) Molecular interplay between mammalian target of rapamycin (mTOR), amyloid-beta, and Tau: effects on cognitive impairments. J Biol Chem 285: 13107–13120. 20178983 10.1074/jbc.M110.100420 PMC2857107

[11] Chauhan A, Sharma U, Jagannathan NR, Reeta KH, Gupta YK (2011) Rapamycin protects against middle cerebral artery occlusion induced focal cerebral ischemia in rats. Behav Brain Res 225: 603–609. 21903138 10.1016/j.bbr.2011.08.035

[12] Cimarosti H, Henley JM (2008) Investigating the mechanisms underlying neuronal death in ischemia using in vitro oxygen-glucose deprivation: potential involvement of protein SUMOylation. Neuroscientist 14: 626–636. 19029060 10.1177/1073858408322677 PMC3310903

[13] Hermann DM, Matter CM (2007) Tissue plasminogen activator-induced reperfusion injury after stroke revisited. Circulation 116: 363–365. 17646593 10.1161/CIRCULATIONAHA.107.712380

[14] Liebeskind DS (2009) Reperfusion for acute ischemic stroke: arterial revascularization and collateral therapeutics. Curr Opin Neurol 23: 36–45. 10.1097/WCO.0b013e328334da32 19926989

[15] Sharp ZD, Strong R (2010) The role of mTOR signaling in controlling mammalian life span: what a fungicide teaches us about longevity. J Gerontol A Biol Sci Med Sci 65: 580–589. 20083554 10.1093/gerona/glp212

[16] Li W, Petrimpol M, Molle KD, Hall MN, Battegay EJ, et al (2007) Hypoxia-induced endothelial proliferation requires both mTORC1 and mTORC2. Circ Res 100: 79–87. 17110594 10.1161/01.RES.0000253094.03023.3f

[17] Copp J, Manning G, Hunter T (2009) TORC-specific phosphorylation of mammalian target of rapamycin (mTOR): phospho-Ser2481 is a marker for intact mTOR signaling complex 2. Cancer Res 69: 1821–1827. 19244117 10.1158/0008-5472.CAN-08-3014 PMC2652681

[18] Ma XM, Blenis J (2009) Molecular mechanisms of mTOR-mediated translational control. Nat Rev Mol Cell Biol 10: 307–318. 19339977 10.1038/nrm2672

[19] Arsham AM, Neufeld TP (2006) Thinking globally and acting locally with TOR. Curr Opin Cell Biol 18: 589–597. 17046229 10.1016/j.ceb.2006.09.005

[20] Hara K, Yonezawa K, Weng QP, Kozlowski MT, Belham C, et al (1998) Amino acid sufficiency and mTOR regulate p70 S6 kinase and eIF-4E BP1 through a common effector mechanism. J Biol Chem 273: 14484–14494. 9603962 10.1074/jbc.273.23.14484

[21] Sengupta S, Peterson TR, Sabatini DM (2010) Regulation of the mTOR complex 1 pathway by nutrients, growth factors, and stress. Mol Cell 40: 310–322. 20965424 10.1016/j.molcel.2010.09.026 PMC2993060

[22] Yuan Y, Guo Q, Ye Z, Pingping X, Wang N, et al (2010) Ischemic postconditioning protects brain from ischemia/reperfusion injury by attenuating endoplasmic reticulum stress-induced apoptosis through PI3K-Akt pathway. Brain Res 1367: 85–93. 20940001 10.1016/j.brainres.2010.10.017

[23] Xu Y, Zhang Q, Yu S, Yang Y, Ding F (2010) The protective effects of chitooligosaccharides against glucose deprivation-induced cell apoptosis in cultured cortical neurons through activation of PI3K/Akt and MEK/ERK1/2 pathways. Brain Res 1375: 49–58. 21167818 10.1016/j.brainres.2010.12.029

[24] Zhao H (2009) Ischemic postconditioning as a novel avenue to protect against brain injury after stroke. J Cereb Blood Flow Metab 29: 873–885. 19240739 10.1038/jcbfm.2009.13 PMC2736291

[25] Niizuma K, Endo H, Chan PH (2009) Oxidative stress and mitochondrial dysfunction as determinants of ischemic neuronal death and survival. J Neurochem 109 Suppl 1133–138. 19393019 10.1111/j.1471-4159.2009.05897.x PMC2679225

[26] Chen J, Zheng XF, Brown EJ, Schreiber SL (1995) Identification of an 11-kDa FKBP12-rapamycin-binding domain within the 289-kDa FKBP12-rapamycin-associated protein and characterization of a critical serine residue. Proc Natl Acad Sci U S A 92: 4947–4951. 7539137 10.1073/pnas.92.11.4947 PMC41824

[27] Kim DH, Sarbassov DD, Ali SM, King JE, Latek RR, et al (2002) mTOR interacts with raptor to form a nutrient-sensitive complex that signals to the cell growth machinery. Cell 110: 163–175. 12150925 10.1016/s0092-8674(02)00808-5

[28] Oshiro N, Yoshino K, Hidayat S, Tokunaga C, Hara K, et al (2004) Dissociation of raptor from mTOR is a mechanism of rapamycin-induced inhibition of mTOR function. Genes Cells 9: 359–366. 15066126 10.1111/j.1356-9597.2004.00727.x

[29] Dowling RJ, Topisirovic I, Fonseca BD, Sonenberg N (2010) Dissecting the role of mTOR: lessons from mTOR inhibitors. Biochim Biophys Acta 1804: 433–439. 20005306 10.1016/j.bbapap.2009.12.001

[30] Sarbassov DD, Ali SM, Sengupta S, Sheen JH, Hsu PP, et al (2006) Prolonged rapamycin treatment inhibits mTORC2 assembly and Akt/PKB. Mol Cell 22: 159–168. 16603397 10.1016/j.molcel.2006.03.029

[31] Broughton BR, Reutens DC, Sobey CG (2009) Apoptotic mechanisms after cerebral ischemia. Stroke 40: e331–339. 19182083 10.1161/STROKEAHA.108.531632

[32] Matheny RW Jr, Adamo ML (2009) Current perspectives on Akt Akt-ivation and Akt-ions. Exp Biol Med (Maywood) 234: 1264–1270. 19596822 10.3181/0904-MR-138

[33] Rosner M, Hengstschlager M (2008) Cytoplasmic and nuclear distribution of the protein complexes mTORC1 and mTORC2: rapamycin triggers dephosphorylation and delocalization of the mTORC2 components rictor and sin1. Hum Mol Genet 17: 2934–2948. 18614546 10.1093/hmg/ddn192

[34] Spilman P, Podlutskaya N, Hart MJ, Debnath J, Gorostiza O, et al (2010) Inhibition of mTOR by rapamycin abolishes cognitive deficits and reduces amyloid-beta levels in a mouse model of Alzheimer’s disease. PLoS One 5: e9979. 20376313 10.1371/journal.pone.0009979 PMC2848616

[35] Chong ZZ, Shang YC, Zhang L, Wang S, Maiese K (2011) Mammalian target of rapamycin: hitting the bull’s-eye for neurological disorders. Oxid Med Cell Longev 3: 374–391. 10.4161/oxim.3.6.14787 PMC3154047 21307646

[36] Fahling M (2009) Cellular oxygen sensing, signalling and how to survive translational arrest in hypoxia. Acta Physiol (Oxf) 195: 205–230. 18764866 10.1111/j.1748-1716.2008.01894.x

[37] Franke H, Illes P (2006) Involvement of P2 receptors in the growth and survival of neurons in the CNS. Pharmacol Ther 109: 297–324. 16102837 10.1016/j.pharmthera.2005.06.002

[38] Neary JT (2005) Protein kinase signaling cascades in CNS trauma. IUBMB Life 57: 711–718. 16511963 10.1080/15216540500319143

[39] Mullonkal CJ, Toledo-Pereyra LH (2007) Akt in ischemia and reperfusion. J Invest Surg 20: 195–203. 17613695 10.1080/08941930701366471

[40] Stiles BL (2009) PI-3-K and AKT: Onto the mitochondria. Adv Drug Deliv Rev 61: 1276–1282. 19720099 10.1016/j.addr.2009.07.017

[41] Kroczynska B, Kaur S, Platanias LC (2009) Growth suppressive cytokines and the AKT/mTOR pathway. Cytokine 48: 138–143. 19682919 10.1016/j.cyto.2009.07.009

[42] Read DE, Gorman AM (2009) Involvement of Akt in neurite outgrowth. Cell Mol Life Sci 66: 2975–2984. 19504044 10.1007/s00018-009-0057-8 PMC11115732

